# Spinocerebellar ataxia type 17-digenic* TBP/STUB1* disease: neuropathologic features of an autopsied patient

**DOI:** 10.1186/s40478-022-01486-6

**Published:** 2022-12-07

**Authors:** Rie Saito, Yui Tada, Daisuke Oikawa, Yusuke Sato, Makiko Seto, Akira Satoh, Kodai Kume, Nozomi Ueki, Masahiro Nakashima, Shintaro Hayashi, Yasuko Toyoshima, Fuminori Tokunaga, Hideshi Kawakami, Akiyoshi Kakita

**Affiliations:** 1 Departments of Pathology, Brain Research Institute, Niigata University, 1-757 Asahimachi, Chuo-ku, Niigata, 951-8585 Japan; 2 Department of Molecular Epidemiology, Research Institute for Radiation Biology and Medicine, Hiroshima University, 1-2-3 Kasumi, Minami-ku, Hiroshima, 734-8553 Japan; 3 Department of Medical Biochemistry, Graduate School of Medicine, Osaka Metropolitan University, 1- 4-3 Asahi-machi, Abeno-ku, Osaka, 545-8585 Japan; 4 Department of Chemistry and Biotechnology, Graduate School of Engineering, Tottori University, 4-101 Koyama-cho Minami, Tottori, 680-8552 Japan; 5 Section of Neurology, Nagasaki Kita Hospital, 800 Motomurago, Togitsu-cho, Nishisonogi-gun, Nagasaki, 851-2103 Japan; 6 Department of Tumor and Diagnostic Pathology, Atomic Bomb Disease Institute, Nagasaki University Graduate School of Biomedical Sciences, 1-12-4 Sakamoto, Nagasaki, 852-8523 Japan; 7 Department of Neurology, Mishima Hospital, 1713-8, Fujikawa, Nagaoka, Niigata, 940-2302 Japan; 8 Department of Neurology, Brain Disease Center, Agano Hospital, 6317-5, Yasuda, Agano, Niigata, 959- 2221 Japan; 9 Department of Pathology, Brain Research Institute, Niigata University, 1-757 Asahimachi, Chuo-ku, Niigata, 951-8585 Japan

**Keywords:** SCA17-DI, *TBP*, *STUB1*, polyQ stretch, Huntington’s disease-like symptoms, Neuropathology

## Abstract

Spinocerebellar ataxia (SCA) type 17-digenic TBP/STUB1 disease (SCA17-DI) has been recently segregated from SCA17, caused by digenic inheritance of two gene mutations – intermediate polyglutamine-encoding CAG/CAA repeat expansions (polyQ) in *TBP* (*TBP*_41 − 49_) and *STUB1* heterozygosity – the former being associated with SCA17, and the latter with SCA48 and SCAR16 (autosomal recessive). In SCA17, most patients carry intermediate *TBP*_41 − 49_ alleles but show incomplete penetrance, and the missing heritability can be explained by a new entity whereby *TBP*_41 − 49_ requires the *STUB1* variant to be symptomatic. The *STUB1* gene encodes the chaperone-associated E3 ubiquitin ligase (CHIP) involved in ubiquitin-mediated proteasomal control of protein homeostasis. However, reports of the neuropathology are limited and role of *STUB1* mutations in SCA17-DI remain unknown. Here we report the clinicopathologic features of identical twin siblings, one of whom was autopsied and was found to carry an intermediate allele (41 and 38 CAG/CAA repeats) in *TBP* and a heterozygous missense mutation in *STUB1* (p.P243L). These patients developed autosomal recessive Huntington’s disease-like symptoms. Brain MRI showed diffuse atrophy of the cerebellum and T2WI revealed hyperintense lesions in the basal ganglia and periventricular deep white matter. The brain histopathology of the patient shared features characteristic of SCA17, such as degeneration of the cerebellar cortex and caudate nucleus, and presence of 1C2-positive neurons. Here we show that mutant CHIP fails to generate the polyubiquitin chain due to disrupted folding of the entire U box domain, thereby affecting the E3 activity of CHIP. When encountering patients with cerebellar ataxia, especially those with Huntington’s disease-like symptoms, genetic testing for *STUB1* as well as *TBP* should be conducted for diagnosis of SCA17-DI, even in cases of sporadic or autosomal recessive inheritance.

**Supplementary Information:**

The online version contains supplementary material available at 10.1186/s40478-022-01486-6.

## Introduction

To date, although diseases with true digenic inheritance (DI) have been rarely reported, they should be considered when encountering patients showing non-Mendelian inheritance, and broad-spectrum phenotypes such as spinocerebellar degeneration. Spinocerebellar ataxia type 17 (SCA17) is an autosomal dominant cerebellar ataxia characterized by cerebellar ataxia and dementia with sometimes extensive variability in phenotypes such as Huntington’s disease-like symptoms (HDL), caused by abnormal expansion of a CAG/CAA repeat encoding a polyglutamine (polyQ) tract in the *TATA-box binding protein* (*TBP*) gene [1]. It has long been unexplained why the penetrance differs depending on the number of polyQ repeats: ≥49 such repeats being fully penetrant, whereas 41–48 repeats, termed intermediate alleles, are associated with reduced penetrance, and half of heterozygotic individuals in SCA17 families are healthy. A recent genetic study has revealed the pathogenesis of the SCA17/HDL phenotype in which intermediate alleles arise through digenic inheritance of two gene mutations – *TBP* polyQ and a heterozygous *STUB1* variant – the latter being associated with SCA48 and the spinocerebellar autosomal recessive type 16 (SCA17-DI) [2]. Another group has identified heterozygous mutations in *STUB1* with intermediate alleles in *TBP* in patients exhibiting a progressive dementia syndrome similar to frontotemporal dementia, with only mild cerebellar atrophy on MRI [3]. However, reports of the neuropathologic features are limited [4, 5] and role of *STUB1* mutations in SCA17-DI remain unknown. Here, we describe in detail the clinicopathologic features of an autopsied patient with SCA17-DI and demonstrate the possible pathogenicity of STUB1.

## Case presentation

### Patient 1

**Fig. 1:**
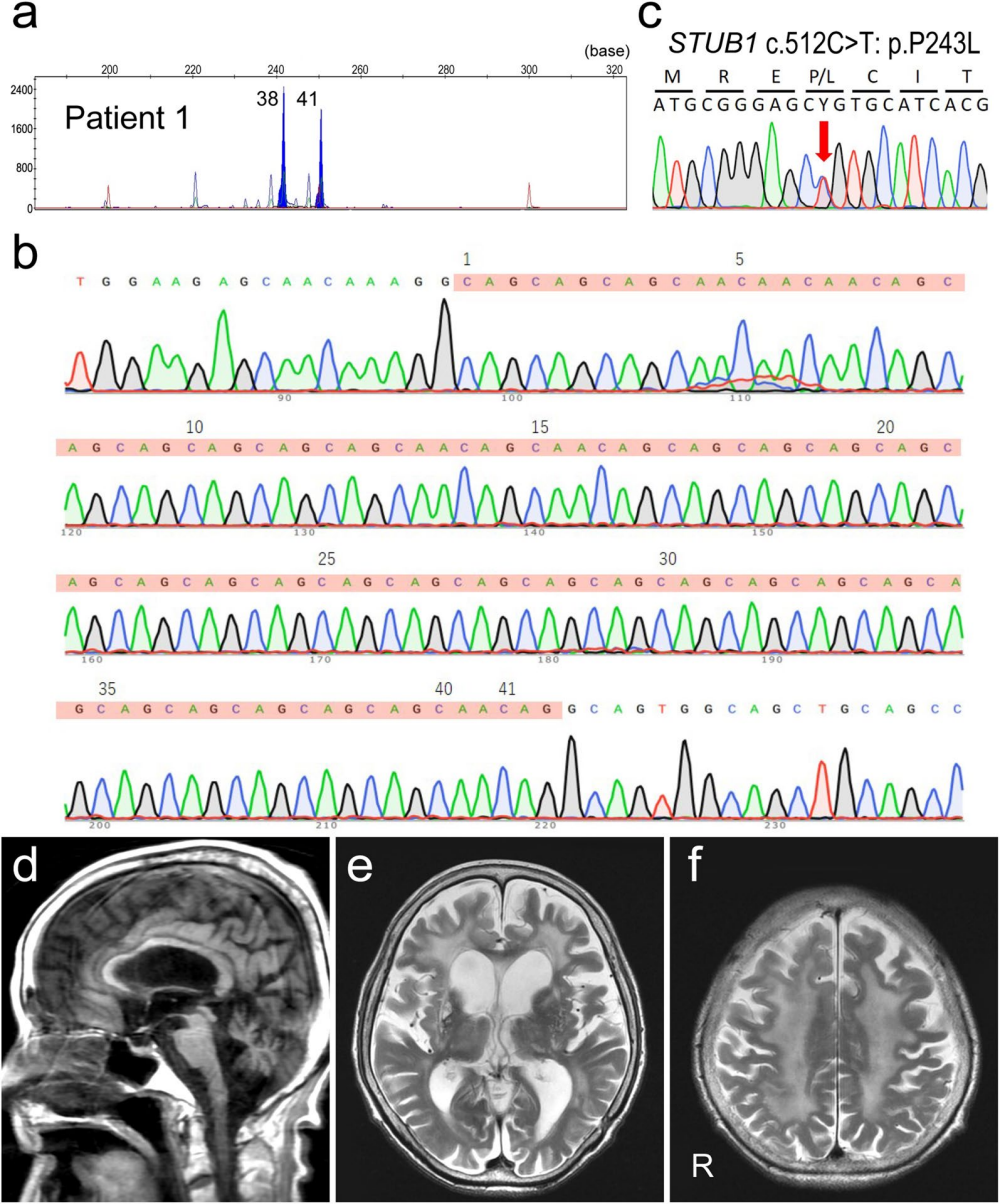
Genetic analysis and MRI findings. **a** Fragment analysis of *TBP*. The numbers besides the peaks indicate the numbers of repeats. **b** Sanger sequencing of the repeat region of the *TBP* gene. The repeat sequences are marked in *light red*. The numbers indicate the numbers of repeats. **c** Sanger sequencing of the variant. *Red arrow* indicates the variant. **d-f** Brain MRI images of patient 2 at the age of 82 years. **d** Severe atrophy of the cerebellum is evident on the T1-weighted sagittal image. **e**, **f** Diffuse cerebral atrophy and lateral ventricular enlargement accompanied by bilateral hyperintense lesions on the T2-weighted image in the basal ganglia, thalamus, and deep white matter. R, the right side of the brain

A 62-year-old Japanese woman from a non-consanguineous family, whose identical twin sister had shown similar symptoms (patient 2), presented with gait disturbance. No other family members showed similar disorders. Their mother had died of a malignant tumor at the age of 72, but no significant ataxia or cognitive impairment had been observed until her death. Their father had been healthy until his 90s. The patient had exhibited normal physical and neurological development. At the age of 68 years, she was admitted to a hospital due to dancing-like involuntary movements in the hands and feet. Neurological examination revealed choreic movement, saccadic eye movement, slurred speech, limb and trunk ataxia, and increased deep tendon reflexes in the upper and lower limbs. Babinski sign was negative. No superficial sensory disturbance or Romberg sign was detected. There was no evidence of bladder or rectal disturbance. Brain MRI revealed severe atrophy of the cerebellum. The cerebrum also showed diffuse atrophy and bilateral hyperintense lesions on T2WI in the basal ganglia and periventricular deep white matter (data not shown). Thus, the patient was diagnosed as having hereditary cerebellar ataxia with leukoencephalopathy, but genetic analysis excluded such diseases including spinocerebellar ataxia type 1 (SCA1) and dentatorubral-pallidoluysian atrophy (DRPLA). Thereafter, her condition slowly deteriorated and she demonstrated cognitive decline. At the age of 73 years, her unsteadiness worsened and she became bedridden. At the age of 76 years, she died of gallbladder cancer. General autopsy was performed, at which time the brain weighed 890 g. Genetic analysis revealed an intermediate allele (41 and 38 CAG/CAA repeats) in *TBP* and a heterozygous missense mutation in *STUB1* (p.P243L) (Fig. 1a–c), establishing a diagnosis of SCA17-DI [2].

### Patient 2

The patient presented with symptoms similar to those of patient 1. She was a 57-year-old Japanese woman with gait disturbance, and five years later she became unable to walk. She had no previous medical history except for surgery for an acoustic tumor at the age of 52 years. At the age of 66 years, she suddenly developed choreic movement similar to those of patient 1. Thus, the patient was thought to have the same hereditary disease, but genetic analysis excluded SCA1 and DRPLA. She became bedridden due to severe trunk ataxia at the age of 69 years, followed by worsening cognitive decline. Brain MRI revealed severe atrophy of the cerebellum and diffuse atrophy of the cerebrum and basal ganglia, showing bilateral hyperintense lesions on T2WI in the basal ganglia, thalamus, and deep white matter (Fig. 1d–f). She then suffered repeated bouts of aspiration pneumonia and died at the age of 88 years. No autopsy or genetic testing for *STUB1* or *TBP* was performed.

### Neuropathologic features (patient 1)

**Fig. 2:**
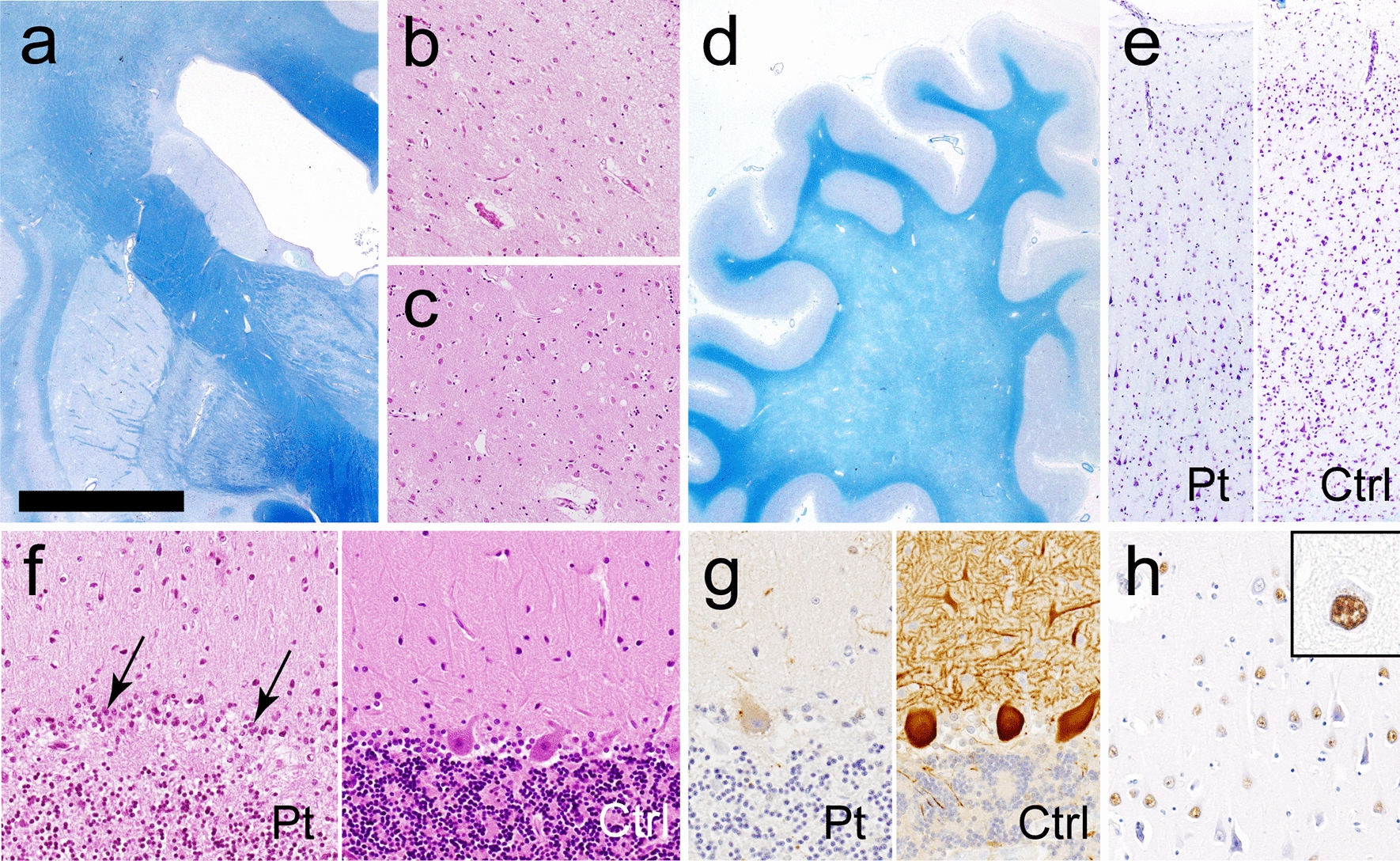
Neuropathologic findings (**a**) Coronal section showing atrophy of the caudate nucleus. Klüver-Barrera staining (KB). **b**, **c** Neuronal loss with gliosis in the basal ganglia: moderate in the caudate nucleus **b** and mild in the putamen (**c**). **d** Diffuse myelin pallor in the frontal white matter. KB. **e** Mild neuronal loss in the frontal cortex, accentuated in the deep cortical layer. KB. **f** Loss of Purkinje cells with Bergmann gliosis (*arrows*) in the cerebellar cortex. HE staining. **g** Depleted immunoreactivity of calbindin-D28k in the cerebellar cortex. The cell body and dendrites of Purkinje cells are strongly stained in the control brain. Calbindin-D28k immunohistochemistry (IHC). **h** Numerous neurons possessing 1C2-positive diffuse staining in nuclei and the magnified image (*inset*). Sector CA1 of Ammon’s horn. 1C2-IHC. Ctrl, control; Pt, patient. Bar in **a** = 1 cm for **a**; 350 μm for **b**, **c**; 1.5 cm for **d**; 400 μm for **e**; 150 μm for **f, h**; and 200 μm for **g**

Macroscopically, atrophy of the basal ganglia was prominent in the caudate nucleus, which showed moderate neuronal loss with gliosis. Neuron reduction was also observed in the deep layer of the frontal and motor cortices, where the white matter showed diffuse myelin pallor (Fig. 2a–e). Severe loss of Purkinje cells and granule cells with Bergman gliosis (Fig. 2f, arrows) were evident (Fig. 2f). Immunoreactivity of calbindin-D28k in the remaining Purkinje cells was depleted (Fig. 2g). The brain showed no pathological features suggestive of complications arising from Alzheimer’s disease (ABC score: A3B1C1) or Parkinson’s disease (Lewy body disease: none). No neuronal loss or focal gliosis was evident in the spinal cord, except for mild loss of neurons in the anterior horns and myelinated fibers in the corticospinal tract. Immunohistochemistry for expanded polyglutamine stretches using 1C2 antibody demonstrated diffuse accumulation in the neuronal nuclei in a diffuse pattern (neuronal intranuclear inclusions: NIIs). NIIs were restricted to the central nervous system, and most frequently detectable in sector CA1 of Ammon’s horn, where 67% of neurons possessed 1C2-positive nuclear inclusions (Fig. 2h). Table 1 summarizes the neuronal loss and distribution of the inclusions.

**Table 1:** Summary of neuropathological findings in the patients with SCA17-DI, SCA17 and SCA48

	SCA17-DI				SCA17				SCA48				
	Present patient		Fujigasaki et al. [4, 5]		Bruni et al. [6]		Toyoshima et al. [7]		Roux et al. [5]	Mol et al. [8]		Chen et al. [9]	
	*TBP*_41/38_/ *STUB1* p.P243L		*TBP*_46/37_/ *STUB1* p.R154C		*TBP*_52/wild_		*TBP*_48/48_		*STUB1* p.A46P	*STUB1* p.C244Y		*STUB1* p.I53T, p.F37L	
	Neuronal loss	NIIs	Neuronal loss	NIIs^a^	Neuronal loss	NIIs	Neuronal loss	NIIs	Neuronal loss	Neuronal loss	NIIs^a,b^	Neuronal loss	NIs^a,c^
*Cerebrum *													
Frontal cortex (Cx/WM^d^)	1/3	1	1	1	1	2	1/2	2	0	0	1	na	na
Motor (Cx/WM^d^)	1/2	2	na	na	2	2	1/1	2	0	0	1	na	na
Temporal (Cx/WM^d^)	0/0	2	1	1	na	na	0/0	2	0	0	1	na	na
Occipital (Cx/WM^d^)	0/0	2	1	1	2	2–3	0/0	2	0	1	1	na	na
*Subcortical area *													
Hippocampus (CA1/CA4/dentate gyrus)	1/0/0	3/0/1	0	1	0	1/0/na	1/0/0	3/2/0	0	na	1	na	na
Amygdala	1	1	na	na	na	na	0	2	na	na	na	na	na
NBM	0	0	na	na	na	na	0	1	na	na	na	na	na
Caudate/putamen	2/1	1/1	0/0	1/0	3/2	1/1	2/2	3/3	0/0	na	1	0	na
Globus pallidus internal /external	0	1/1	0	0	0	1	0/0	1	0	na	1	0	na
Thalamus	0	0	0	0	3^e^	1	1-2^f^	1	na	na	na	na	na
Subthalamic nucleus	0	0	na	na	na	na	1	2	0	3^ g^	na	na	na
*Brainstem *													
Superior colliculus	1	2	na	na	na	na	0	2	na	na^h^	na	na	na
Oculomotor nucleus	0	1	0	0	0	2	0	1	na	na	na	na	na
Red nucleus	0	1	0	0	na	na	0	2	na	na	na	na	na
Substantia nigra	0	1	0	0	1	2	1	2	1	na	1	na	na
Locus ceruleus	0	0	1	0	0	0	0	1	na	na	na	na	na
Pontine nucleus	1	2	0	1	0	2	0	1	0	na	1	na	na
Hypoglossal nucleus	0	0	na	na	na	na	0	1	na	na	1	na	na
Dorsal vagal nucleus	0	1	na	na	na	na	0	1	na	na	na	na	na
IO	2	2	0	0	2	3	1	2	1	na	1	na	na
*Cerebellum *													
Cortex (Purkinje cells/granule cells)	3/2	0/1	3/1	0/0	3/1	0/1	2/1	1/2	3/2	3	1	3/2	1
Dentate nucleus	1	0	0	1	1	2	0	1	0	na	na	na	na
Spinal cord													
Anterior horn (C/L)	1/0	0/1	0	1	0	0	0/0	0/0	na	na	na	na	na
IML	0	1	na	na	na	na	0	0	na	na	na	na	na
Clarke’s column	0	2	na	na	na	na	0	0	na	na	na	na	na
Posterior column^d^	0	na	na	na	na	na	0	na	na	na	na	na	na
Corticospinal tract^d^	1	na	na	na	na	na	0	na	na	na	na	na	na
Spinocerebellar tract^d^	0	na	na	na	na	na	0	na	na	na	na	na	na
Peripheral nerve													
Dorsal root ganglion	0	0	na	na	na	na	0	0	na	na	na	na	na

The density of inclusions (NIIs) was graded according to the percentage of inclusion-bearing neurons: 0, none; 1, 0–10%; 2, 10–40%; 3, > 40%

Cx, cortex; WM, white matter; NBM, nucleus basalis of Meynert; IO, inferior olivary nucleus; IML, intermediolateral nucleus; NIIs, neuronal intranuclear inclusions; NIs, neuronal inclusions; na, not available; ^a^ No information on the density of NIIs was available, and was described as either present (1) or absent (0); ^b^ The inclusions were detected by antibodies against p62, ubiquitin, or 1C2 as neuronal intranuclear inclusions or cytoplasmic inclusions; ^c^ Ubiquitinated inclusions; ^d^ Myelin pallor; ^e^ Dorso-medial nucleus, medial nuclei, nucleus reuniens; ^f^ Medial and centromedial nucleus; ^g^ Gliosis; ^h^ The description was limited to severe neuronal loss in the mesencephalon and medulla oblongata

To investigate STUB1 (protein) alteration in the affected brain, we performed immunohistochemistry using an antibody against SUTB1. In a previous pathologic study, aberrant STUB1 localization was demonstrated in the distal PJC dendrites of patients with SCA48, while STUB1 was immunoreactive in somatodendrites in the control [9]. However, our analysis revealed no such difference in localization between them (Supplementary Fig. 1 in Additional file 1).

### E3 activity of the CHIP-p.P243L mutant

**Fig. 3:**
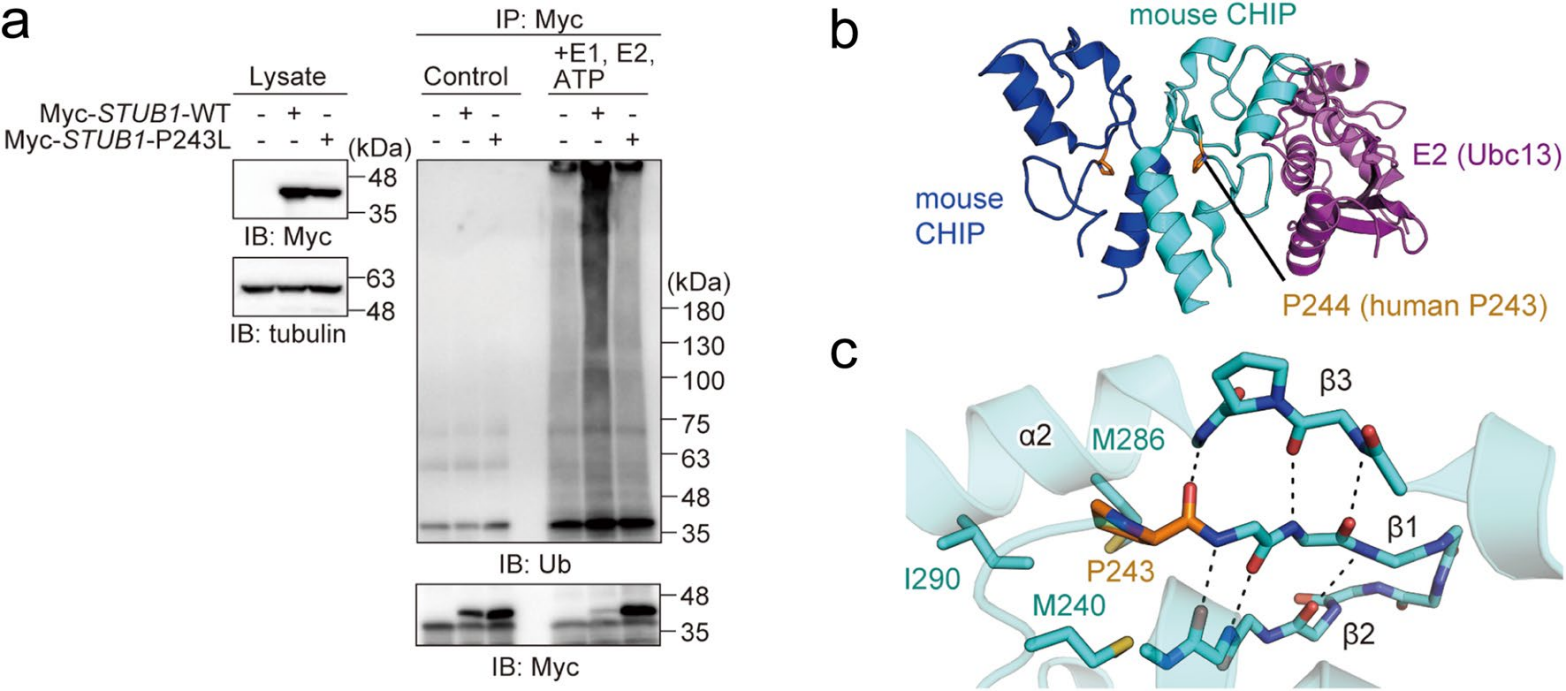
Attenuating effect of p.P243L on E3 activity in CHIP. **a** Impaired E3 activity of the S*TUB1*-p.P243L mutant. The WT or p.P243L mutant of Myc-STUB1 was transiently expressed in HEK293T cells. Then, the anti-Myc immunoprecipitates were subjected to in vitro ubiquitination assay, and samples were immunoblotted with the indicated antibodies. **b** Crystal structure of the mouse CHIP U box dimer in complex with E2 (Ubc13 and Uev1a) (PDB: 2c2v). One protomer in the CHIP dimer is colored cyan and the other blue. Ubc13 is colored magenta. P243 in the human CHIP (P244 in mouse STUB1) is shown in orange. **c** Close-up view around the β-sheet of the CHIP U box (PDB: 2c2v). The β-sheet is shown as sticks. The backbone hydrogen bonds in the β-sheet are indicated by dashed lines. Side chains other than proline on the β-sheet are omitted. The residue numbers indicated are those of human CHIP

We then investigated whether the *STUB1* mutation caused a functional change in the encoded protein, chaperone-associated E3 ubiquitin ligase (CHIP), which is involved in the ubiquitin-mediated proteasomal control of protein homeostasis, and is known to facilitate degradation of misfolded proteins in neurodegenerative diseases [10]. Therefore, we assessed the effect of *STUB1* p.P243L mutation on E3 ubiquitin ligase activity by transiently expressing the wild type (WT) or p.P243L mutant of *STUB1* in HEK293T cells, followed by immunoprecipitation and in vitro ubiquitination assay (Fig. 3a). In the presence of E1, E2 (UbcH5a), and ubiquitin, *STUB1*-WT efficiently generated the polyubiquitin chain, whereas the p.P243L mutant failed. These results clearly indicated that p.P243L polymorphism in the U box domain affects the E3 activity of CHIP. Details of methods are in Additional file 1.

## Discussion and conclusions

We have described the clinicopathologic features of patients harboring an intermediate allele (41 and 38 CAG/CAA repeats) in *TBP* and a heterozygous missense mutation in *STUB1* (p.P243L), and demonstrated reduced E3 ubiquitin ligase activity of the *STUB1*-p.P243L mutant.

The clinical features of the present identical twins were quite similar to each other, with onset of ataxic gait at around 60 years of age, followed by chorea and cognitive decline, and a period of approximately 10 years from onset to becoming bedridden, although patient 1 had half the disease duration of patient 2 due to cancer. Reflecting their clinical course, brain MRI also showed features in common. Similarly, the clinical presentation in both patients resembled that of two previously reported cases harboring an intermediate allele (41 and 37, and 43 and 41 CAG/CAA repeats, respectively) in *TBP* and the same heterozygous missense mutation in *STUB1* (p.P243L) [2]: all of the patients were female and demonstrated cerebellar ataxia and cognitive decline, and three of the four developed chorea with a Huntington’s disease-like (HDL) phenotype. On the other hand, these three families showed different inheritance patterns, the present family showing autosomal recessive inheritance and the other two families autosomal dominant inheritance and a sporadic pattern, being consistent with a previous report of complex forms of inheritance in SCA17-DI [2]. The difference in age at disease onset between the present patients and two other reported patients (around 60 years for the former and 30s for the latter) is also within the wide onset age range observed for SCA17-DI as a whole [2].

The pathological findings in the present patient were similar to those reported previously for SCA17-DI [4, 5] and SCA17 [6, 7] in that most patients exhibited degeneration of the cerebellar cortex and striatum with the presence of 1C2-positive neurons showing diffuse nuclear staining [4, 6, 7] (Table 1). Only in one of these cases, the striatum including the caudate nucleus, which is known to be associated with choreic movement in Huntington’s disease, was not affected and indeed no involuntary movements were noted [4] (Supplementary table 2 in Additional file 1). Overall, the histopathological alterations may be slightly milder in SCA17-DI than in SCA17, and more cases will need to be studied to clarify the difference.

Given the similarities of pathology between SCA17-DI and SCA17, despite the fact that the former had a mutation in *STUB1* whereas the latter did not, a major question naturally arises as to whether a heterozygous *STUB1* mutation alone could affect the phenotype. There have been a few reports on the neuropathology of SCA48, which has both commonalities and differences relative to SCA17-DI and SCA17. The reports on SCA48 have highlighted severe degeneration of the cerebellar cortex [5, 8, 9]. On the other hand, no alterations were observed within the striatum in two of those reports [5, 9] (Table 1), despite the fact that one of the two patients presented with HDL showing chorea and dystonia [5]. In terms of 1C2-immunoreactive structures, one had scattered 1C2-positive neuronal intranuclear inclusions [8], while the other did not [5]. The *TBP* repeat size in those patients was not stated. Even considering that the specificity of the 1C2 antibody could be sometimes unstable, it would be of importance to determine the repeat size of *TBP* in patients with heterozygous *STUB1* mutation in order to better understand the role of heterozygous *STUB1* mutation.

It has been postulated that the pathogenicity of mutations in *STUB1* centers on E3 activity of CHIP [10], as we demonstrated, but details of the pathomechanism have remained unclear. The E3 activity of six SCAR16-associated *STUB1* variants – p.E28K, p.N65S, p.K145Q, p.M211I, p.S236T, and p.T246M – have been evaluated, and it has been reported that p.T246M mutation in the U box affects the structure and E3 activity of CHIP [11]. In contrast, another U box mutant, p.S236T, exhibited E3 activity equivalent to that of the wild type, suggesting that mutations within the U box domain, a Zn-free E3 active site similar to the RING finger domain, may or may not significantly affect E3 activity depending on the mutated residue [11]. As we identified reduced E3 activity of CHIP resulting from p.P243L mutation, we therefore further analyzed the effect of *STUB1* p.P243L on the conformation of the U box domain using data from the deposited crystal structure (Fig. 3b, c) [12]. A previous report has indicated that the proline residue corresponding to P243 in human CHIP is highly conserved in all U box proteins in mammals [13]. P243 in human CHIP (P244 in mouse CHIP) is not directly involved in CHIP dimerization or binding to E2 (Fig. 3b). The CHIP U box domain contains three β strands (β1–3), and P243 is located at the end of β1, which seems to promote β-sheet termination and folding of the U box domain (Fig. 3c). However, in the p.P243L mutant, the NH group of L243 may form a hydrogen bond with the N-terminal main chain of an α-helix (α2), which extends the C-terminal end of β3. Because β3 is immediately followed by an α2, the extension of β3 would inhibit α2 helix formation and disrupt overall folding of the U box (Fig. 3c). Moreover, P243 in human CHIP forms a hydrophobic core with M240, M286, and I290 (M241, L287, I291 in mouse CHIP) to stabilize the structure of the U box [14], and p.P243L mutation disrupts these interactions (Fig. 3c). Together, these results suggest that the p.P243L mutation disrupts the folding of the entire U box domain, and impairs ubiquitin ligase activity, leading to insufficient degradation of TATA box-binding protein with moderately expanded poly-Q tracts and disease onset.

In conclusion, we have presented the second genetically confirmed autopsy case of SCA17-DI presenting with a Huntington’s disease-like phenotype, and have demonstrated the functional and conformational changes resulting from *STUB1* mutation associated with ubiquitin ligase activity. Further clinicopathologic and molecular studies are needed to clarify how *TBP* polyQ and *STUB1* mutations interact and affect the phenotypic variability of SCA17-DI.

## Supplementary Information

**Additional file 1: Figure S1.** STUB1 immunohistochemistry in the patient. **Table S1.** Primary antibodies. **Table S2.** Summary of the clinical features in the autopsied patients with SCA17-DI, SCA17, and SCA48.

## Data Availability

The datasets used and analysed during this study are available from the corresponding author on reasonable request.

## Abbreviations

*TBP*: TATA box-binding protein
SCA17-DI: Spinocerebellar ataxia type 17-digenic *TBP*/*STUB1* disease
SCA: Spinocerebellar ataxia
PolyQ: Abnormal expansion of a CAG/CAA repeat encoding a polyglutamine
SCAR: Spinocerebellar autosomal recessive
HDL: Huntington’s disease-like symptoms
NIIs: Neuronal intranuclear inclusions.

## References

[1] Toyoshima Y, Onodera O, Yamada M, Tsuji S, Takahashi H (2021) Spinocerebellar ataxia type 17. In: Adam MP, Ardinger HH, Pagon RA, eds et al (1993) GeneReviews. University of Washington

[2] Magri S, Nanetti L, Gellera C, Sarto E, Rizzo E, Mongelli A (2022). Digenic inheritance of STUB1 variants and TBP polyglutamine expansions explains the incomplete penetrance of SCA17 and SCA48. Genet Med.

[3] Reis MC, Patrun J, Ackl N, Winter P, Scheifele M, Danek A (2022). A severe dementia syndrome caused by intron retention and cryptic splice site activation in STUB1 and exacerbated by TBP repeat expansions. Front Mol Neurosci.

[4] Fujigasaki H, Martin JJ, De Deyn PP, Camuzat A, Deffond D, Stevanin G (2001). CAG repeat expansion in the TATA box-binding protein gene causes autosomal dominant cerebellar ataxia. Brain.

[5] Roux T, Barbier M, Papin M, Davoine CS, Sayah S, Coarelli G (2020). Clinical, neuropathological, and genetic characterization of STUB1 variants in cerebellar ataxias: a frequent cause of predominant cognitive impairment. Genet Med.

[6] Bruni AC, Takahashi-Fujigasaki J, Maltecca F, Foncin JF, Servadio A, Casari G (2004). Behavioral disorder, dementia, ataxia, and rigidity in a large family with TATA box-binding protein mutation. Arch Neurol.

[7] Toyoshima Y, Yamada M, Onodera O, Shimohata M, Inenaga C, Fujita N (2004). SCA17 homozygote showing Huntington’s disease-like phenotype. Ann Neurol.

[8] Mol MO, van Rooij JGJ, Brusse E, Verkerk AJMH, Melhem S, den Dunnen WFA (2020). Clinical and pathologic phenotype of a large family with heterozygous STUB1 mutation. Neurol Genet.

[9] Chen DH, Latimer C, Yagi M, Ndugga-Kabuye MK, Heigham E, Jayadev S (2020). Heterozygous STUB1 missense variants cause ataxia, cognitive decline, and STUB1 mislocalization. Neurol Genet.

[10] Ronnebaum SM, Patterson C, Schisler JC (2014). Emerging evidence of coding mutations in the ubiquitin-proteasome system associated with cerebellar ataxias. Hum Genome Var.

[11] Pakdaman Y, Sanchez-Guixé M, Kleppe R, Erdal S, Bustad HJ, Bjørkhaug L (2017). In vitro characterization of six STUB1 variants in spinocerebellar ataxia 16 reveals altered structural properties for the encoded CHIP proteins. Biosci Rep.

[12] Zhang M, Windheim M, Roe SM, Peggie M, Cohen P, Prodromou C (2005). Chaperoned ubiquitylation–crystal structures of the CHIP U box E3 ubiquitin ligase and a CHIP-Ubc13-Uev1a complex. Mol Cell.

[13] Hatakeyama S, Yada M, Matsumoto M, Ishida N, Nakayama KI (2001). U box proteins as a new family of ubiquitin-protein ligases. J Biol Chem.

[14] Andersen P, Kragelund BB, Olsen AN, Larsen FH, Chua NH, Poulsen FM (2004). Structure and biochemical function of a prototypical Arabidopsis U-box domain. J Biol Chem.

