# Supplementary material for: Spinocerebellar ataxia type 17-digenic TBP/STUB1 disease: neuropathologic features of an autopsied patient
Source: Acta Neuropathol Commun. 2022 Dec 7;10:177. doi: 10.1186/s40478-022-01486-6 (PMC9727856; doi:10.1186/s40478-022-01486-6)
Supplement: Supplementary file 1 — Additional file 1: Figure S1. STUB1 immunohistochemistry in the patient. Table S1. Primary antibodies. Table S2. Summary of the clinical features in the autopsied patients with SCA17-DI, SCA17, and SCA48. [file 40478_2022_1486_MOESM1_ESM.pdf]

# Additional file 1

*Acta Neuropathologica Communications*

## Spinocerebellar ataxia type 17-digenic *TBP*/*STUB1* disease: autopsy features of an autopsied patient

**Rie Saito[1], Yui Tada[2], Daisuke Oikawa[3], Yusuke Sato[4], Makiko Seto[5], Akira Satoh[5], Kodai Kume[2], Nozomi Ueki[6], Masahiro Nakashima[6], Shintaro Hayashi[1,7], Yasuko Toyoshima[1,8], Fuminori Tokunaga[3], Hideshi Kawakami[2], Akiyoshi Kakita[1]**

[1]Departments of Pathology, Brain Research Institute, Niigata University, 1-757 Asahimachi, Chuo-ku, Niigata 951-8585, Japan.

[2]Department of Epidemiology, Research Institute for Radiation Biology and Medicine, Hiroshima University, 1-2-3 Kasumi, Minami-ku, Hiroshima 734-8553, Japan.

[3]Department of Medical Biochemistry, Graduate School of Medicine, Osaka Metropolitan University, 1-4-3 Asahi-machi, Abeno-ku, Osaka 545-8585, Japan

[4]Department of Chemistry and Biotechnology, Graduate School of Engineering, Tottori University, 4-101 Koyama-cho Minami, Tottori 680-8552, Japan.

[5]Section of Neurology, Nagasaki Kita Hospital, 800 Motomurago, Togitsu-cho, Nishisonogi-gun, Nagasaki 851-2103, Japan.

[6]Department of Tumor and Diagnostic Pathology, Atomic Bomb Disease Institute, Nagasaki University Graduate School of Biomedical Sciences, 1-12-4 Sakamoto, Nagasaki 852-8523, Japan.

[7]Department of Neurology, Mishima Hospital, 1713-8 Fujikawa, Nagaoka, Niigata 940-2302, Japan.

[8]Department of Neurology, Brain Disease Center, Agano Hospital, 6317-5 Yasuda, Agano, Niigata 959-2221, Japan.

Correspondence to: Akiyoshi Kakita, MD, PhD
Department of Pathology, Brain Research Institute, Niigata University, 1-757 Asahimachi, Chuo-ku, Niigata 951-8585, Japan.
TEL: +81-25-2270673
FAX: +81-25-2270817
E-mail: kakita@bri.niigata-u.ac.jp

**Supplementary methods**
**Supplementary figure 1:** STUB1 immunohistochemistry in the patient
**Supplementary references**
**Supplementary table 1:** Primary antibodies
**Supplementary table 2:** Summary of the clinical features in the autopsied patients with SCA17-DI, SCA17, and SCA48.

## Supplementary methods

### *Histopathologic analysis*

The brain and spinal cord were fixed with 10% buffered formalin, and multiple tissue blocks were embedded in paraffin. Histological examination was performed on 4-µm-thick sections using hematoxylin and eosin and Klüver-Barrera staining. In addition, selected sections were immunostained with antibodies against calbindin D-28k, STUB1 (protein) [2], polyglutamine and p62 (**Supplementary table 1**). Antibodies against amyloid β 11-28, phosphorylated tau and phosphorylated α-synuclein were used to assess senile pathologic changes based on the "ABC" score [7] and the fourth consensus report of the DLB consortium [5]. Bound antibodies were visualized by the peroxidase-polymer-based method using a Histofine Simple Stain MAX-PO kit (Nichirei, Tokyo, Japan) with diaminobenzidine as the chromogen. Immunostained sections were counterstained with hematoxylin.

### *Genetic analysis*

Genomic DNA was extracted from the fresh-frozen frontal cortex of the present patient, and also from the occipital cortex of the patient who was homozygotic for SCA17 [10].

**Exome sequencing**: The extracted genomic DNA was used to prepare an exome library using a BGI platform sequencer. Details of the method were described in our previous report [4]. Exome capture and library preparation were performed using xGenTM Exome Research Panel v2 (IDT, Coralville, USA). Whole-exome sequencing was performed using a BGI platform sequencer (DNBSEQ-G400). We identified a heterozygous missense mutation in *STUB1* (p.P243L), which was validated by Sanger sequencing (**Fig. 1c**). To evaluate the pathogenicity of the variants, we employed the following criteria: (1) two or more prediction algorithms (SIFT, PP2, Mutation Taster, and CADD) were positive (the CADD score threshold was set to $>20$) and (2) the allele frequency of the variant was $\leq 0.005$.

**Fragment analysis**: The *TBP* repeat region was amplified by PCR using an ExTaq (Takara, Japan) based on our previous report [~~5~~8]. The primer sequences were: Forward 5'-(6-FAM) CCTTATGGCACTGGACTGA and Reverse 5'-GTTCCCTGTGTTGCCTGCTG. The amplicon size was measured using an ABI PRISM 3130xl Genetic Analyzer (Applied Biosystems, USA). The size standard was GeneScan™-500 ROX (Applied Biosystems, USA).

**Sanger sequencing**: *STUB1* exon 6 was amplified by PCR using an Expand High Fidelity PCR System (Roche, Switzerland). The primer sequences were: Forward 5'-GAAGAGGAAGGTGAGTGTGTGTC, Reverse 5'-CAGAGATGAATGCGTCAATAACC. The *TBP* repeat region was amplified using the same method as that for fragment analysis. The amplicons were cloned into pMD20 (TAKARA, Japan) using a TA cloning method. Cycle sequencing of the amplicons was performed using the BigDye Terminator v3.1 Cycle Sequencing

Kit (Applied Biosystems, USA). The sequence of amplicons was analyzed by the ABI PRISM 3130xl Genetic Analyzer (Applied Biosystems, USA).

***Plasmids***

The open reading frame of human *STUB1* was amplified by reverse-transcription PCR. P243L mutation was introduced by the QuikChange method, and the nucleotide sequences were verified. The coding sequences were tagged with Myc, amplified by PCR, and cloned into the pcDNA3.1 expression vector (Invitrogen).

***Cell culture and transfection***

HEK293T cells (ATCC) were cultured in DMEM containing 10% fetal bovine serum, 100 IU/ml penicillin G, and 100 μg/ml streptomycin at 37°C under 5% $CO_2$. Transfection experiments were performed using polyethyleneimine (PEI MAX; Polysciences).

***In vitro ubiquitination assay***

For in vitro ubiquitination assay, HEK293T cells were transfected with pcDNA3-Myc-*STUB1*-WT or p.P243L mutant. After 24 h, the cells were lysed with lysis buffer containing 20 mM Tris-HCl, pH 7.5, 150 mM NaCl, 1% Triton X-100, and 1 mM DTT, and immunoprecipitated using rabbit anti-Myc antibody and protein A beads (Cytiva). After a wash with lysis buffer, the beads were incubated with 5 μg/ml recombinant mouse His-E1, 20 μg/ml E2 (recombinant human UbcH5a; Boston Biochem), and 250 μg/ml ubiquitin (Sigma-Aldrich), in buffer containing 10 mM Tris-HCl, pH 7.5, 5 mM $MgCl_2$, 1 mM DTT, and 2 mM ATP at 30 °C for 4 h. Then, the samples were subjected to SDS–PAGE, followed by immunoblotting.

***SDS-PAGE and immunoblotting***

Samples were separated by SDS-PAGE using 2/15% gradient gels (Cosmobio) and transferred to PVDF membranes. After blocking the membranes in Tris-buffered saline containing 0.1% Tween-20 (TBS-T) with 5% skim milk for 2 h at room temperature (RT), they were incubated overnight with the appropriate primary antibodies diluted in TBS-T containing 5% skim-milk at 4°C. The following antibodies were used for immunoblotting analyses: Myc (HRP-Conjugate), ubiquitin, and tubulin. For immunoprecipitation, rabbit anti-Myc antibody was used (**Supplementary table 1**). Then, the membranes were incubated with anti-mouse IgG horseradish peroxidase-conjugated secondary antibody (NA931V; 1:10,000; Cytiva) diluted in TBS-T containing 5% skim-milk for 1.5 h at RT. For detection, SuperSignal West Pico PLUS (34577; Thermo Fisher Scientific) or Luminata Forte (WBLUF0100; Millipore) was used. The chemiluminescent images were obtained with a Fusion Solo S imaging system (Vilber).

**Supplementary figure 1. STUB1 immunohistochemistry in the patient.**

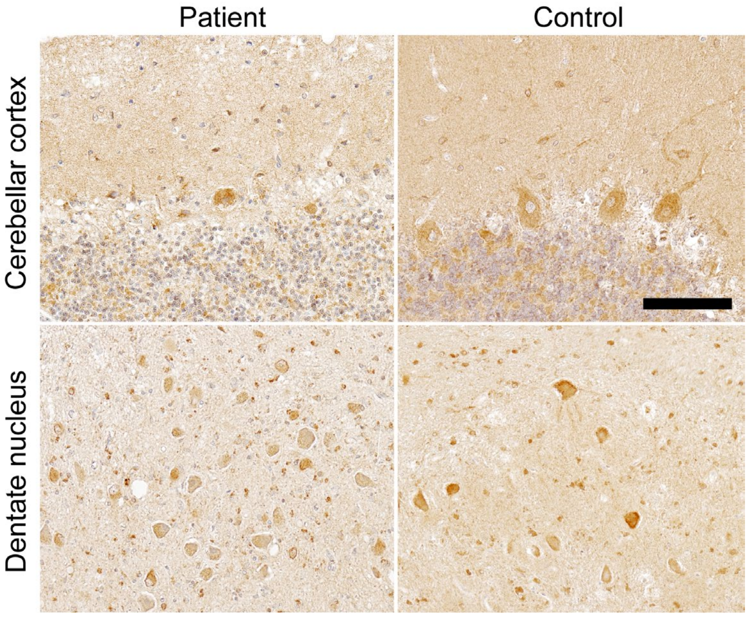


Images of the Purkinje cell layer (affected lesion) and dentate nucleus (unaffected area). The patient and control display positive immune-reactivity in the cytoplasm and dendrites of the Purkinje cells, and cytoplasm and neurites in the dentate nucleus. Bar = 100 µm in the *upper panels*; 150 µm in the *lower panels*

## Supplementary references

1. Bruni AC, Takahashi-Fujigasaki J, Maltecca F, Foncin JF, Servadio A, Casari G et al (2004). Behavioral disorder, dementia, ataxia, and rigidity in a large family with TATA box-binding protein mutation. Arch Neurol 61:1314-20
2. Chen DH, Latimer C, Yagi M, Ndugga-Kabuye MK, Heigham E, Jayadev S et al (2020) Heterozygous *STUB1* missense variants cause ataxia, cognitive decline, and STUB1 mislocalization. Neurol Genet 6:1-13
3. Fujigasaki H, Martin JJ, De Deyn PP, Camuzat A, Deffond D, Stevanin G et al (2001). CAG repeat expansion in the TATA box-binding protein gene causes autosomal dominant cerebellar ataxia. Brain 124:1939-47
4. Matsuda Y, Morino H, Miyamoto R, Kurashige T, Kume K, Mizuno N et al (2020) Biallelic mutation of *HSD17B4* induces middle age-onset spinocerebellar ataxia. Neurol Genet 6:e396.
5. McKeith IG, Boeve BF, Dickson DW, Halliday G, Taylor JP, Weintraub D et al (2017) Diagnosis and management of dementia with Lewy bodies: fourth consensus report of the DLB Consortium. Neurology 89:88-100
6. Mol MO, van Rooij JGJ, Brusse E, Verkerk AJMH, Melhem S, den Dunnen WFA et al (2020). Clinical and pathologic phenotype of a large family with heterozygous STUB1 mutation. Neurol Genet 6:e417
7. Monine TJ, Phelps CH, Beach TG, Bigio EH, Cairns NJ, Dickson DW et al (2012) National Institute on Aging-Alzheimer's association guidelines for the neuropathologic assessment of Alzheimer's disease: a practical approach. Acta Neuropathol 123:1-11
8. Oda M, Maruyama H, Komure O, Morino H, Terasawa H, Izumi Y et al (2004) Possible reduced penetrance of expansion of 44 to 47 CAG/CAA repeats in the TATA-binding protein gene in spinocerebellar ataxia type 17. Arch Neurol 61:209-212
9. Roux T, Barbier M, Papin M, Davoine CS, Sayah S, Coarelli G et al (2020). Clinical, neuropathological, and genetic characterization of STUB1 variants in cerebellar ataxias: a frequent cause of predominant cognitive impairment. Genet Med 22:1851-62
10. Toyoshima Y, Yamada M, Onodera O, Shimohata M, Inenaga C, Fujita N et al (2004) SCA17 homozygote showing Huntington's disease-like phenotype. Ann Neurol 55:281-286

**Supplementary table 1. Primary antibodies.**

| Antigen (clone) | Antibody species | Source | Dilution | Antigen retrieval |
|---|---|---|---|---|
| Immunohistochemistry | | | | |
| Calbindin-D28k (CB300) | Mouse | Swant, Fribourg, Switzerland | 1:50 | Microwave |
| STUB1 | Rabbit | Abcam, Cambridge, UK | 1:800 | Autoclave (pH 9) |
| STUB1 | Mouse | Proteintech, Rosemont, IL, USA | 1:200 | Autoclave (pH 9) |
| p62 (3/P62) | Mouse | BD, San Jose, CA, USA | 1:1000 | Microwave |
| Polyglutamine (5TF1-1C2) | Mouse | Chemicon, Temecula, CA, USA | 1:16000 | Formic acid |
| Amyloid β (11-28, 12B2) | Mouse | IBL, Gunma, Japan | 1:50 | Formic acid |
| Phosphorylated tau (AT8) | Mouse | Fujirebio, Ghent, Belgium | 1:200 | None |
| Phosphorylated α-synuclein (pSyn#64) | Mouse | Wako, Saitama, Japan | 1:1000 | Formic acid |
| Immunoblotting | | | | |
| Myc (M192-7) | Mouse | MBL, Tokyo, Japan | 1:10000 | None |
| Ubiquitin (P4D1) | Mouse | Santa Cruz Biotechnology, Dallas, TX, USA | 1:1000 | None |
| Tubulin (CLT9002) | Mouse | Cedarlane, Ontario, Canada | 1:3000 | None |
| Immunoprecipitation | | | | |
| Myc (562) | Rabbit | MBL, Tokyo, Japan | 1:500 | None |

**Supplementary table 2. Summary of the clinical features in the autopsied patients with SCA17-DI, SCA17, and SCA48.**

| Disorder | Patient | *TBP* allele | *STUB1* mutation | Sex | Age at onset (years) | Disease duration (years) | Cerebellar ataxia | Cognitive impairment | Psychiatric disorder | Rigidity | Involuntary movement | Epilepsy |
|---|---|---|---|---|---|---|---|---|---|---|---|---|
| SCA17-DI | This case | 41/38 | p.P243L | F | 62 | 14 | Yes | Yes | No | No | Chorea | No |
| SCA17-DI | Fujigasaki et al. [3, 9] | 46/37 | p.R154C | F | 55 | 10 | Yes | Yes | No | Yes | No | No |
| SCA17 | Bruni et al. [1] | 52/wild | na | F | 17 | 30 | Yes | Yes | Yes | Yes | Dystonia, myoclonus | Yes |
| SCA17 | Toyoshima et al. [10] | 48/48 | No | M | 39 | 10 | Yes | Yes | Yes | No | Chorea, ballism | No |
| SCA48 | Roux et al. [9] | na | p.A46P | F | 47 | 15 | Yes | Yes | Yes | Yes | Chorea, dystonia | Yes |
| SCA48 | Mol et al. [6] | na | p.C244Y | na | 65 | 18 | Yes | Yes | No | na | No | No |
| | | | p.C244Y | na | 61 | 10 | Yes | Yes | No | na | Subtle | No |
| | | | p.C244Y | na | 50 | 14 | Yes | Yes | Yes | na | Subtle | No |
| SCA48 | Chen et al. [2] | na | p.I53T | M | 40s | <20 | Yes | Yes | No | No | Short jerky movements | No |
| | | | p.I53T | M | 60s | <19 | Yes | Yes | No | na | No | No |
| | | | p.I53T | F | NA | 10-20? | Yes | Yes | Yes | No | No | No |
| | | | p.F37L | F | 40 | 21 | Yes | Yes | Yes | na | Tremor | No |
